# Supplementary material for: A pan-cancer atlas of somatic mutations in miRNA biogenesis genes
Source: Nucleic Acids Res. 2021 Jan 6;49(2):601–20. doi: 10.1093/nar/gkaa1223 (PMC7826265; doi:10.1093/nar/gkaa1223)
Supplement: gkaa1223_Supplemental_Files [file gkaa1223_supplemental_files.zip › GalkaMarciniak_Suppl_Table S7.docx]

**Supplementary Table S7: The list of miRNAs well recognized in cancer that were differentiated by different types of *DICER1* mutations.**

| miRNA | change | miRNA role in cancer |
| --- | --- | --- |
| miR-200c-5p | downregulated | Recognized as a tumor suppressor miR |
| miR-98-5p | downregulated | Recognized as a tumor suppressor miR |
| let‑7d‑5p and let-7e-5p | downregulated | Members of tumor suppressor miR let-7 family |
| miR-500a-5p | downregulated | Recognized as a tumor suppressor miR |
| miR-7-5p | downregulated | Recognized as a tumor suppressor miR |
| miR-190a-5p | downregulated | Recognized as both an oncogene miR and tumor suppressor miR in various human cancers |
| miR-339-5p | downregulated | Recognized as a tumor suppressor miR |
| miR-188-5p | downregulated | Recognized as a tumor suppressor miR |
| miR-18a-5p | downregulated | Recognized as both an oncogene miR and tumor suppressor miR in various human cancers |
| miR-30d-3p | upregulated | Recognized as oncogene miR |
| miR-30c-3p | upregulated | Passenger strand of oncogene miR-30d-5p |
| miR-219-3p | upregulated | Passenger strand of tumor suppressor miR-219-5p |
| miR-20a-3p | upregulated | Passenger strand of tumor suppressor miR-20a-5p |
| miR-93-3p | upregulated | Passenger strand of oncogene miR-93-5p |
| miR-98-3p | upregulated | Passenger strand of tumor suppressor miR -98-5p |
| miR-181b-3p | upregulated | Passenger strand of miR-181b-5p which is recognized as both oncogene miR and tumor suppressor miR in various human cancers |
| miR-16-2-3p | upregulated | Recognized as a tumor suppressor miR and passenger strand of tumor suppressor miR-16-5p |
| miR-144-3p | upregulated | Recognized as both an oncogene miR and tumor suppressor miR in various human cancers |
| miR-431-3p | upregulated | Recognized as both an oncogene miR and tumor suppressor miR in various human cancers |
