# Supplementary material for: A pan-cancer atlas of somatic mutations in miRNA biogenesis genes
Source: Nucleic Acids Res. 2021 Jan 6;49(2):601–20. doi: 10.1093/nar/gkaa1223 (PMC7826265; doi:10.1093/nar/gkaa1223)
Supplement: gkaa1223_Supplemental_Files [file gkaa1223_supplemental_files.zip › SUPPLEMENTARY MATERIALS_RI.pdf]

SUPPLEMENTARY MATERIALS

A pan-cancer atlas of somatic mutations in miRNA biogenesis genes

Paulina Galka-Marciniak, Martyna Olga Urbanek-Trzeciak, Paulina Maria Nawrocka and Piotr Kozlowski

Supplementary Tables:

Supplementary Table S1: Genomic coordinates of 29 miRNA biogenesis genes (hg38).

Supplementary Table S2: Summary of TCGA samples and mutations in the panel of miRNA biogenesis genes.

Supplementary Table S3: The mutations identified in the panel of miRNA biogenesis genes in the Pan-Cancer.

Supplementary Table S4: Overmutation of the particular miRNA biogenesis genes in specific cancer types (p-values).

Supplementary Table S5: Changes in miRNAs expression in a different group of mutations in *SMAD4*, *SMAD2*, and *DICER1* genes, according to sheet names.

Supplementary Table S6: List of pathways enriched in the genes regulated by the group of miRNAs that are differentiated by mutations in *SMAD4* and *DICER1* (miRPath v3.0), according to sheet names.

Supplementary Table S7: The list of miRNAs well recognized in cancer that were differentiated by different types of *DICER1* mutations.

Supplementary Table S8: Changes in isomiRs levels (5'end-defined main miRNAs) in UCEC samples with the *DICER1* mutations.

Supplementary Figures:

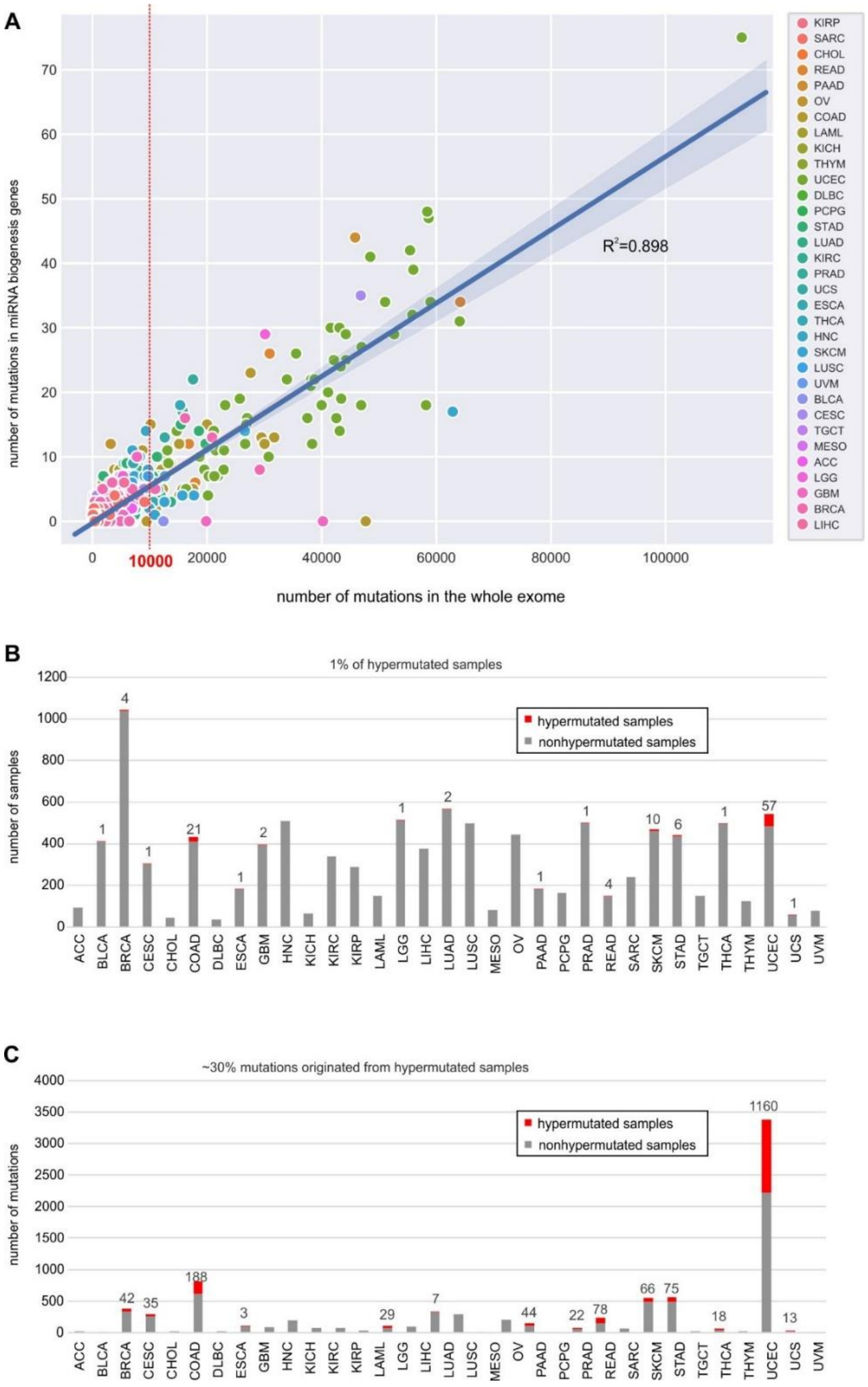

Supplementary Figure S1. Mutations in hypermutated samples. A) Correlation of the number of mutations in the panel of miRNA biogenesis genes (y-axis) and the general burden of mutations (number of mutations in the whole exome; x-axis) in individual cancer samples. The red vertical line indicates the threshold for hypermutated samples. Cancer types are indicated by different colors. B) The proportions of hypermutated (red bars) and nonhypermutated (gray bars) samples (y-axis) in each cancer type (x-axis). C) The proportions of mutations in hypermutated (red bars) and nonhypermutated (gray bars) samples (y-axis) in each cancer type (x-axis).

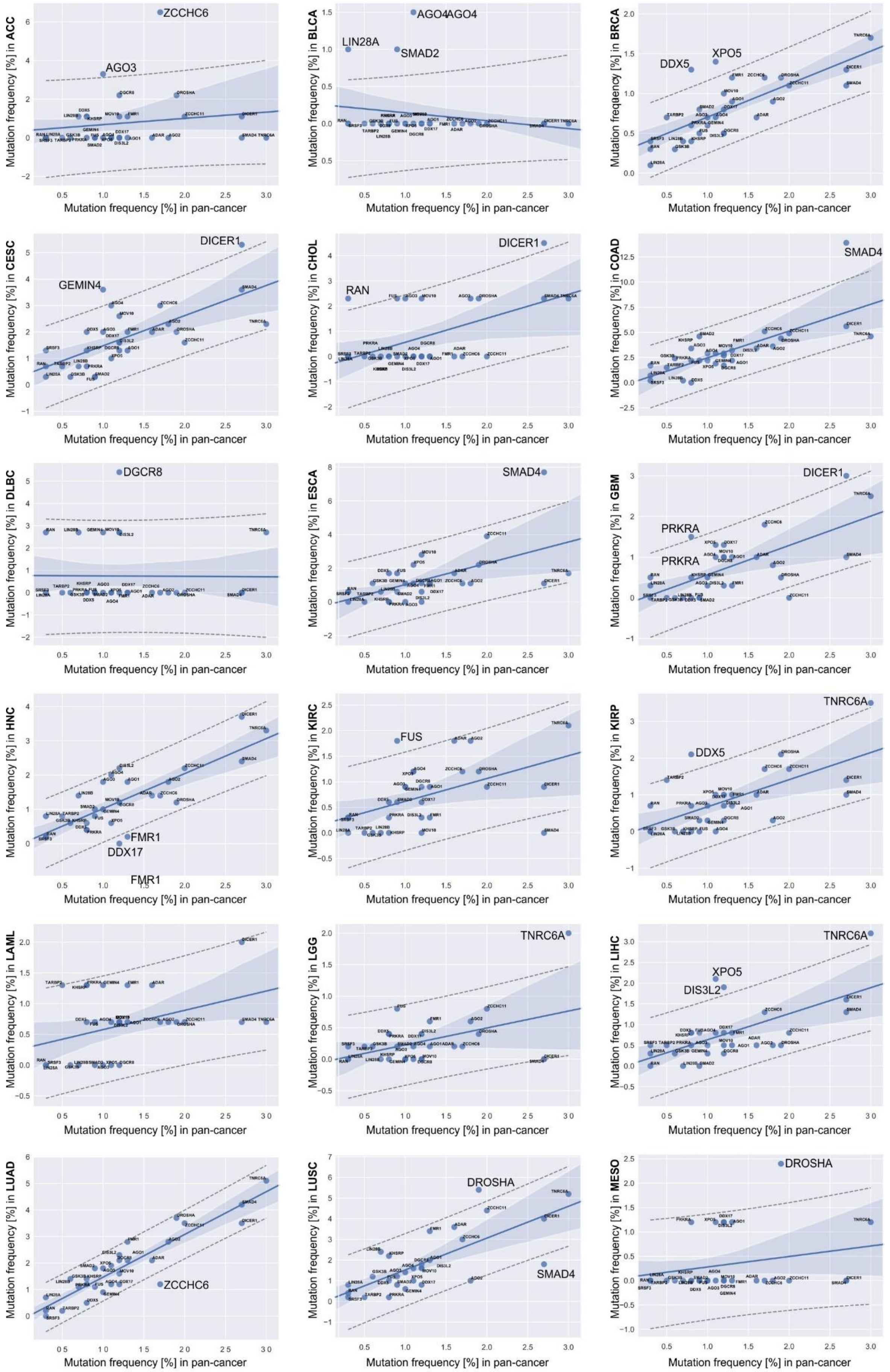

continued on next page

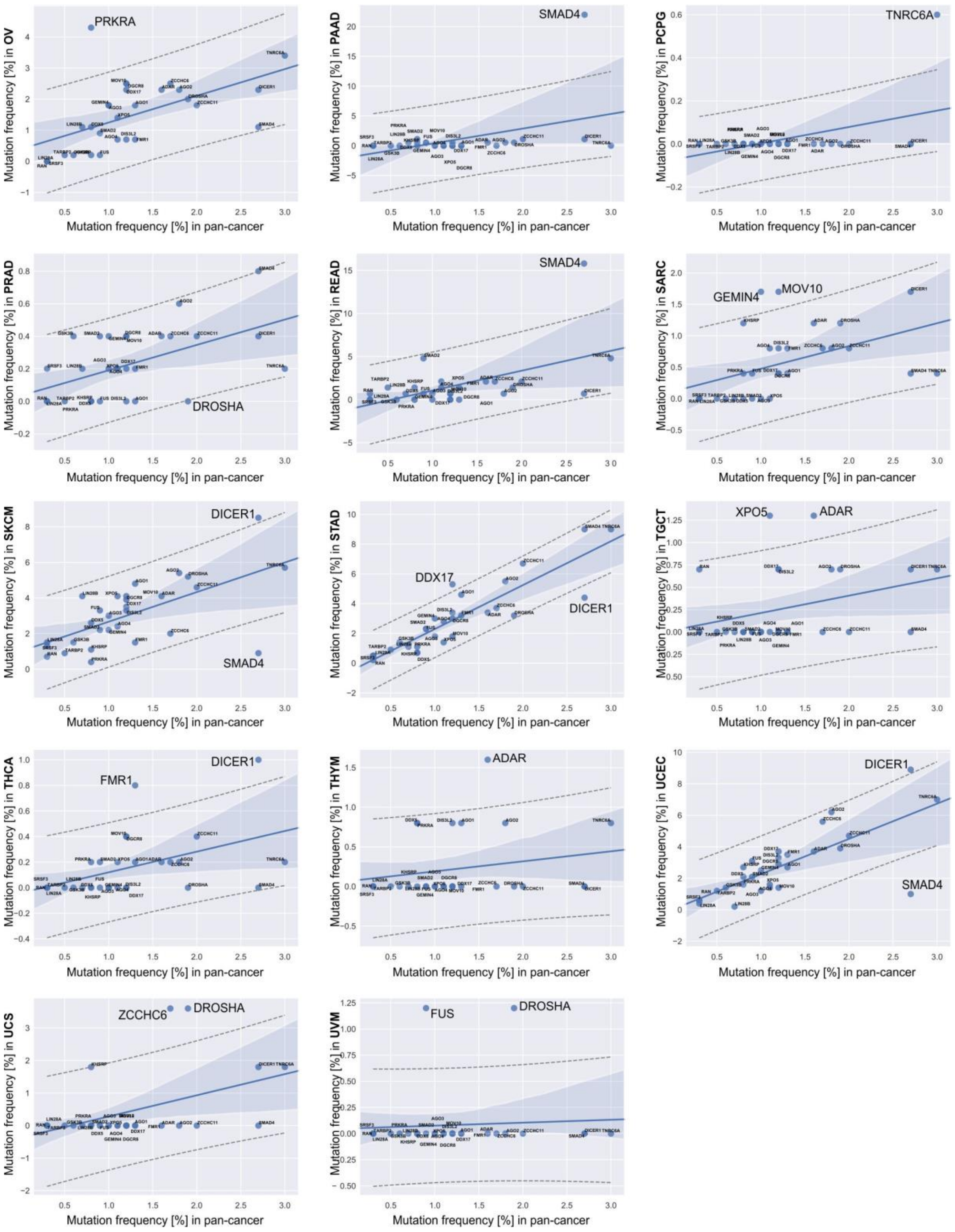

Supplementary Figure S2 Scatterplots showing the correlation between the frequencies of mutations in the miRNA biogenesis genes observed in the individual cancer types (y-axis) and in the remaining pan-cancer (x-axis) samples. In each graph, dots indicating the particular miRNA biogenesis genes, the regression line, and dashed lines representing the confidence interval with 95% probability are shown. Note that the informativeness of some of the graphs is low due to the very low number of mutations identified in some cancer types.

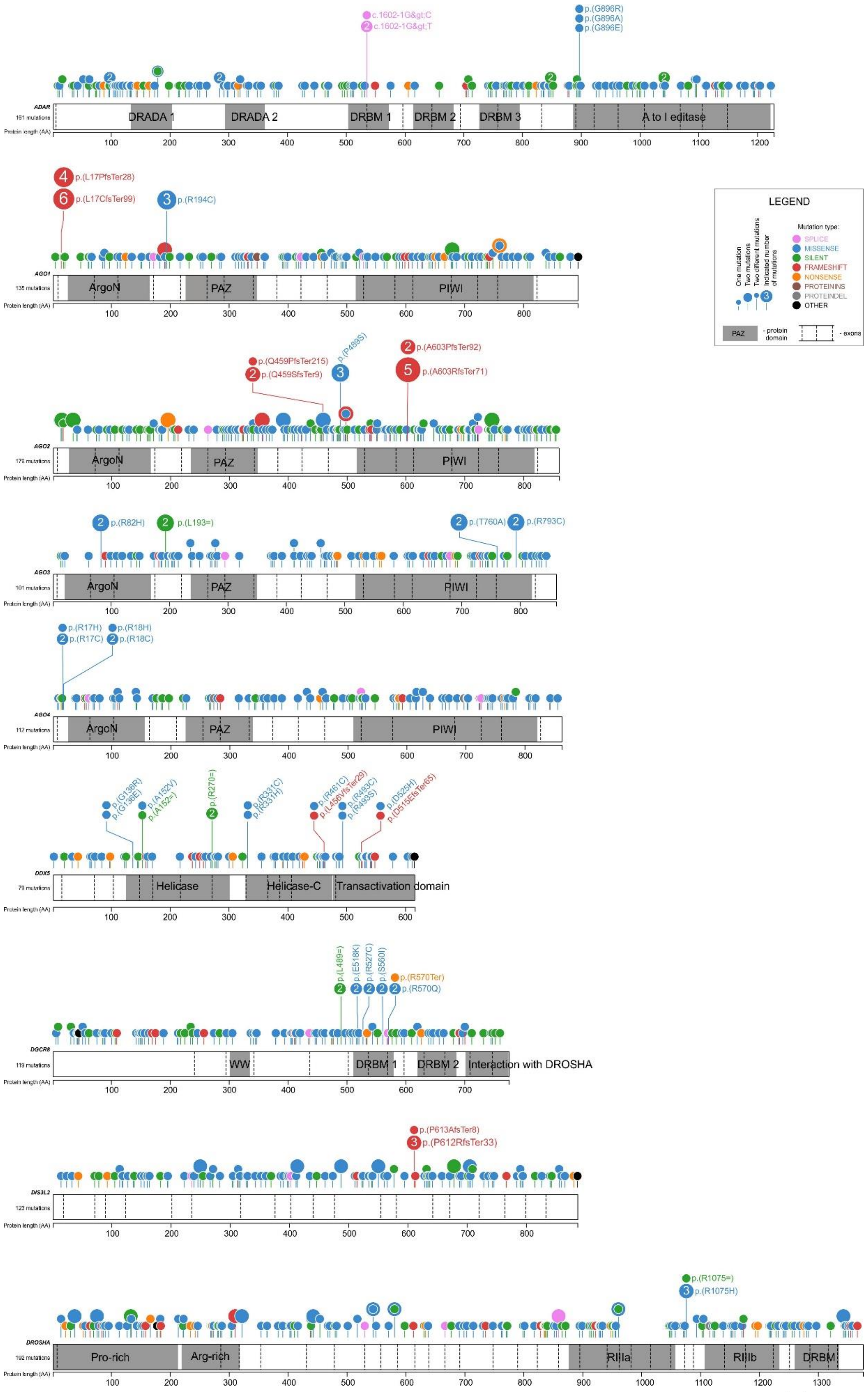

continued on next page

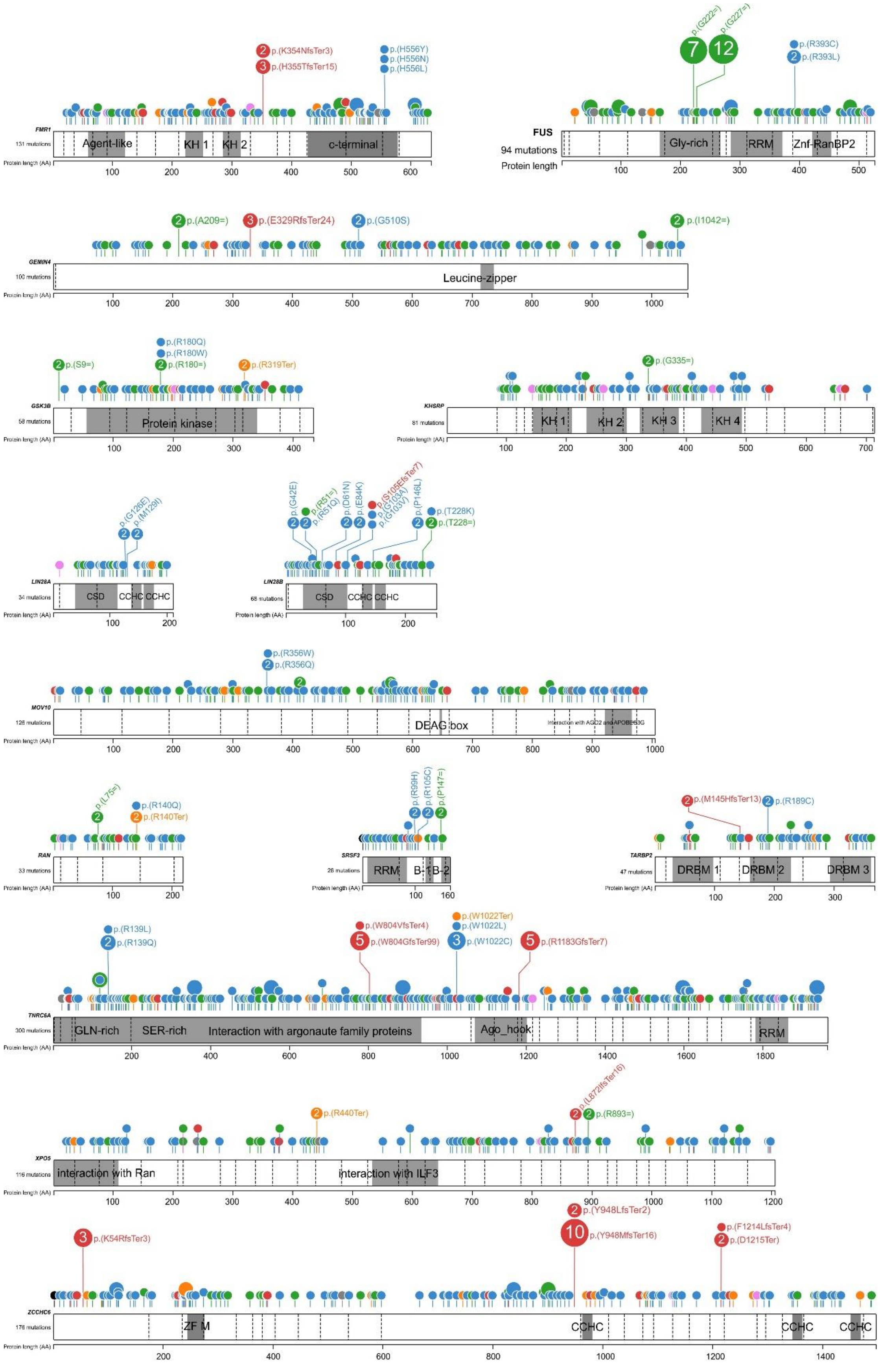

Supplementary Figure S3 Distribution of the identified mutations in the miRNA biogenesis genes. The scheme of the figure is as shown in Figure 3.

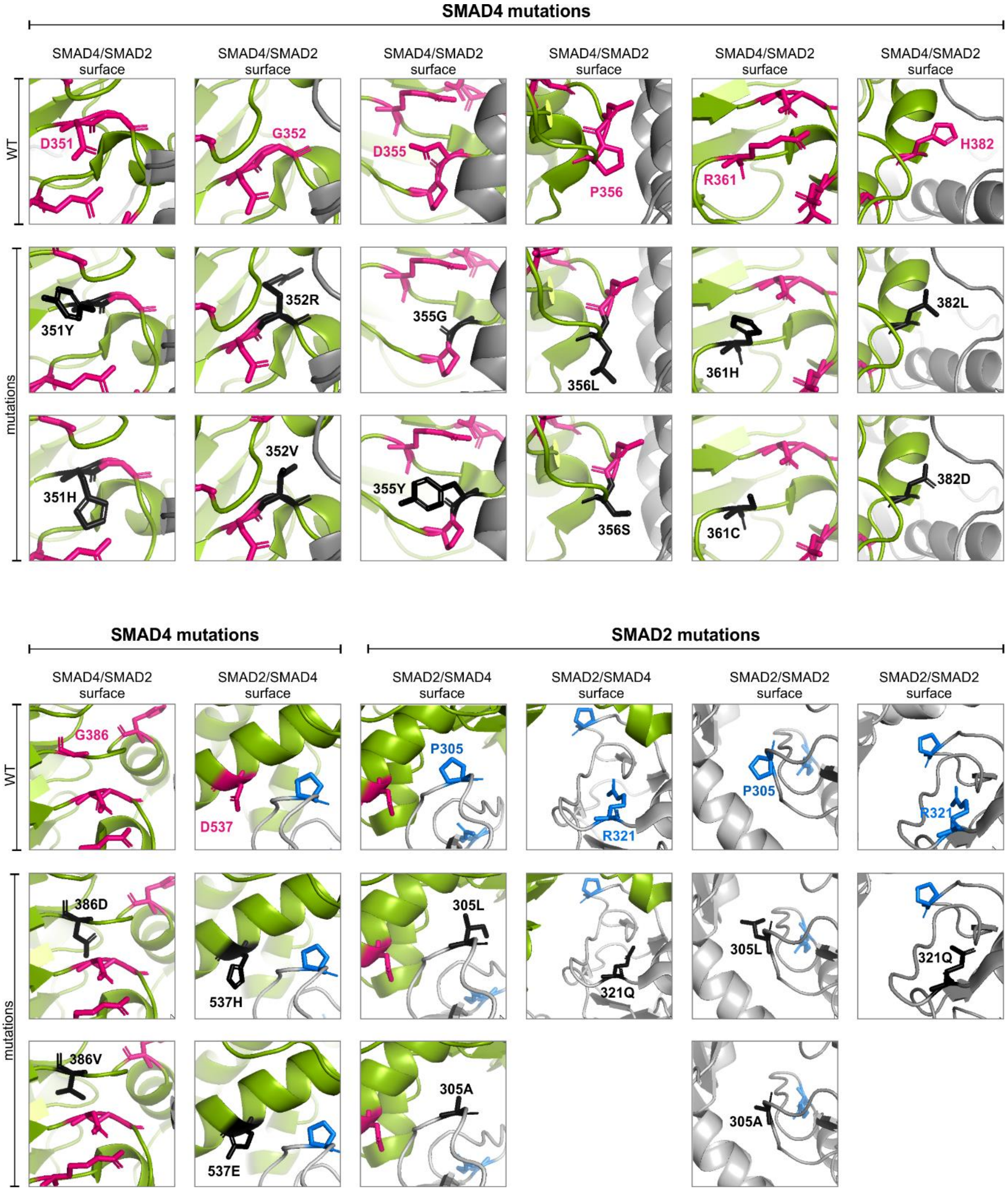

Supplementary Figure S4 The localizations of the most frequent mutations in the SMAD4:(SMAD2)2 heterotrimeric complex in SMAD2/SMAD4, SMAD4/SMAD2, and SMAD2/SMAD2 surfaces (as indicated in Figure 4B). Wild type AAs in mutated residues are indicated in pink (SMAD4) and blue (SMAD2) whereas corresponding mutant AAs are indicated in black.

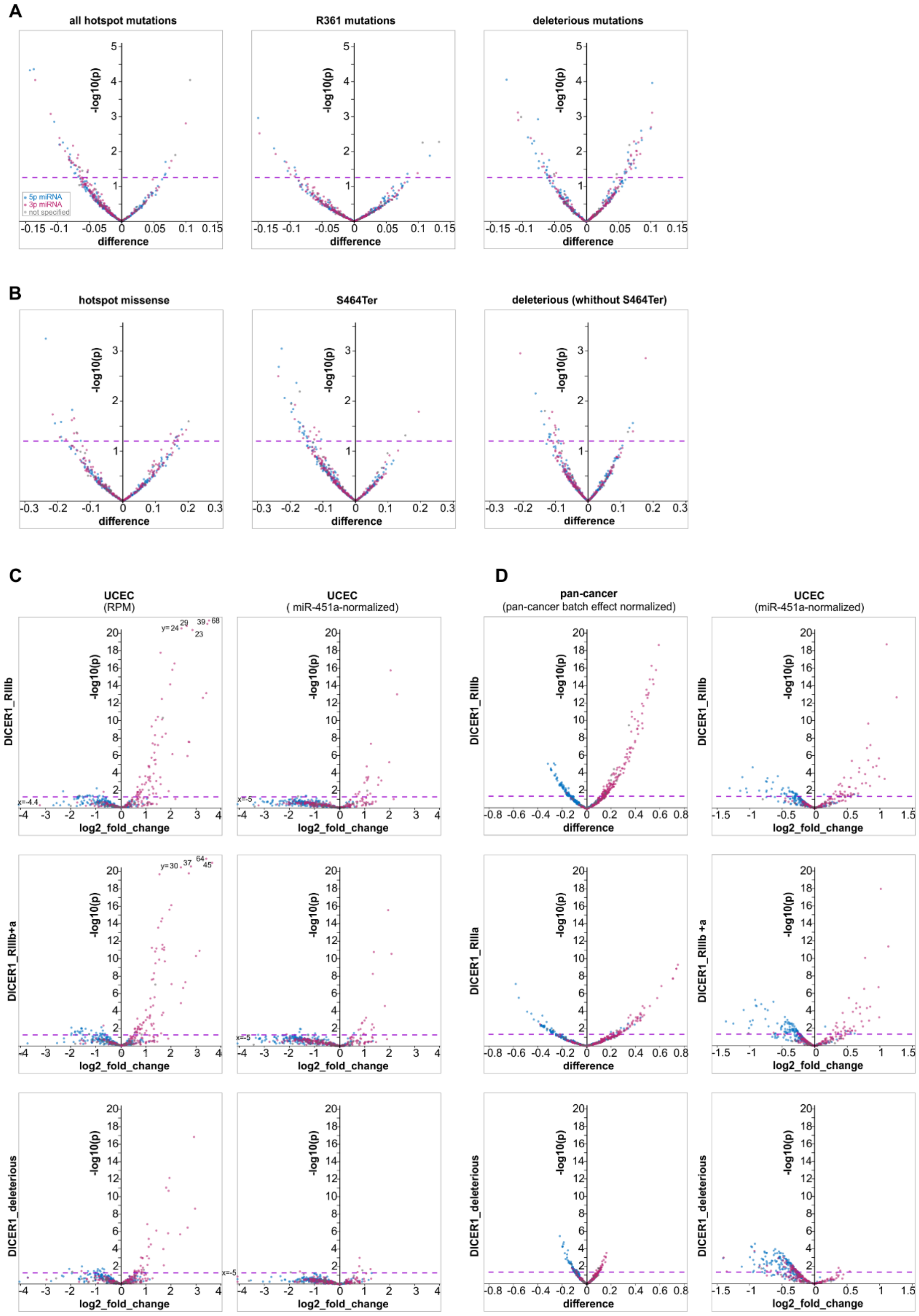

Supplementary Figure S5 Volcano plots depicting miRNA level alterations in samples with different types of *SMAD4* (A), *SMAD2* (B), and *DICER1* (C and D) mutations (indicated above the graphs) compared to samples without any mutations in the particular genes. Panel A and B correspond to Figure 4D and Figure 5A, respectively, but show analysis limited only to high-confidence miRNAs annotated in MirGeneDB. Panel C) corresponds to Figure 6B (2nd and 3rd columns) with the exception that the analyzed input miRNA levels were expressed in RPM but not transformed by batch-effects normalization. Panel D) corresponds to Figure 6B (1st and 3rd columns) but shows analysis limited only to high-confidence miRNAs annotated in MirGeneDB. The scheme of the volcano plots is as shown in Figure 4.
